# Safflower (*Carthamus tinctorius* L.) Response to Cadmium Stress: Morpho-Physiological Traits and Mineral Concentrations

**DOI:** 10.3390/life13010135

**Published:** 2023-01-03

**Authors:** Murat Tunçtürk, Younes Rezaee Danesh, Rüveyde Tunçtürk, Erol Oral, Solmaz Najafi, Lütfi Nohutçu, Arshad Jalal, Carlos Eduardo da Silva Oliveira, Marcelo Carvalho Minhoto Teixeira Filho

**Affiliations:** 1 Department of Field Crops, Faculty of Agriculture, Van Yüzüncü Yıl University, 65090 Van, Türkiye; 2 Department of Plant Protection, Faculty of Agriculture, Urmia University, Urmia 5756151818, Iran; 3 Department of Plant Protection, Rural Engineering and Soils (DEFERS), São Paulo State University (UNESP), Ilha Solteira 01049-010, SP, Brazil

**Keywords:** ascorbate peroxidase, cadmium, malondialdehyde, safflower, growth indices, mineral content

## Abstract

Cadmium is a widely distributed heavy metal in agricultural soils that affects plant growth and productivity. In this context, the current study investigated the effects of different cadmium (Cd) doses (0, 25, 50, 75, and 100 mg L^−1^ of CdSO_4_) on the growth and physiological attributes of safflower (*Carthamus tinctorius* L.) including plant height (cm), root length (cm), fresh weight (g) of root, stem, and leaves, leaf number, macro and micro-nutrients, Se, and heavy metal (Cd, Cr, and Pb) content. The experiment was carried out in a completely randomized design (CRD) with four replicates. The results showed that Cd stress significantly negatively affected all growth indices, macro- and micro-nutrients, and heavy metal content. In addition, it increased the MDA and APX activities. The highest amounts of Fe, Mn, Ni, Pb, Zn, K, Na, Cd, Cr, and Cu were determined in plant roots, while the highest values of Ca and Mg were detected in plant stem tissues. High Cd doses decreased the content of Ca, K, Mg, Cr, Cu, Fe, Mn, Ni, Pb, Se, and Zn in safflower plant tissues by 45.47%, 39.33%, 79.28%, 68.21%, 37.06%, 66.67%, 45.62%, 50.38%, 54.37%, 33.33% and 65.87%, respectively, as compared to the control treatments.

## 1. Introduction

Safflower (*Carthamus tinctorius* L.) is an important oilseed plant with commercial value and is known by different names around the world, including American saffron, dyer saffron, wild saffron, and Zaferan. It is an annual herb with broad leaves and flowers in yellow, red, orange, white, and cream colors [1]. The average seed oil content varies from 30% to 50%. The oil extracted from safflower seeds is more desirable than other plant oils, such as soybean, sunflower, and corn [2]. In addition, safflower is a rich source of linoleic (omega-6) and oleic (omega-9) acids which can reduce blood cholesterol levels and enhance the cooking taste and nutritional value of this crop [2,3]. Safflower is a multipurpose crop well adapted to marginal regions due to its high growth capability under low agricultural inputs and changing climate scenarios [4]. The deep penetrating roots system and short drought sensitivity duration (flower formation to the middle of the grain filling stage) of safflower increase its drought tolerance capacity and cultivation in marginal soils. It is a well-known drought-tolerant plant [5,6]. Safflower is exposed to different geographical conditions, including heavy metal stress. Among the latter, cadmium (Cd) affects this crop’s growth, physiology, and yield traits.

Plants can easily take up cadmium due to its mobility and solubility in soil and water [7]. Several chelating agents carry Cd, which enters groundwater and pollutes drinking and irrigation water, thus causing severe threats to agricultural soils and production [8]. Cadmium has become one of the most devastating environmental issues due to its natural occurrence in the earth’s crust, and presence in synthetic fertilizers, sewage wastes, and atmospheric deposits, etc., linked to anthropogenic human activities [9,10]. Excessive use of phosphorous fertilization contaminates agricultural soils due to the presence of high Cd content that disturbs the food chain [11]. Hyper-accumulation of Cd and other heavy metals in the soil generates severe phytotoxicity that alters plants’ biochemical and physiological processes [12].

Scientific studies on the toxic effect of Cd are increasing day by day. Most of these studies have been focused on the physiological responses against toxicity and general tolerance mechanisms; even a low concentration of 3 mg kg^−1^ of Cd causes soil toxicity [13]. It has 2–20 times more toxic effects than other heavy metals [14]. Cadmium absorbed by plants disrupts several metabolic activities, such as protein synthesis, nitrogen and carbohydrate metabolism, enzyme activation, photosynthesis, and chlorophyll synthesis [7,15]. In addition, cadmium enhances the formation of reactive oxygen species, causing oxidative destruction of thylakoid membrane lipids and inhibiting chlorophyll synthesis, thus reducing nitrate reductase activities and the nitrate assimilation rate of plants [16]. Safflower cultivation has gained importance in regions of intense industrialization and high environmental and soil pollution. However, there needs to be more research on the morpho-physiological aspects and mineral concentration of safflower in Cd-contaminated soils. In this context, it was necessary to determine the performance of safflower, which has been reported as a high Cd accumulator under harsh climatic conditions and Cd-stressed soils. Therefore, this study aimed to determine the effects of Cd on the growth and physiological performance of safflower. In addition, the effects of Cd doses on macro- and micro-nutrient concentration and heavy metals concentration were determined in the root, stem, and leaves of safflower.

## 2. Materials and Methods

### 2.1. Plant and Soil Materials

The safflower cultivar Balcı (cadmium tolerant cultivar) was obtained from previous field studies conducted at Van Yüzüncü Yıl University. Soil analysis was carried out at the Department of Soil Science and Plant Nutrition, Faculty of Agriculture, Van Yüzüncü Yıl University, following the methodology of Özbek et al. [17]. The soil was classified as saline (0.03%), loamy (14.8%), and slightly alkaline (pH = 8.12) with low organic matter (0.96%) and phosphorus and potassium content (1.49 and 6.74 kg ha^−1^, respectively).

### 2.2. Study Location and Experiment Set Up

The research was carried out in a fully controlled plant growth cabinet at the Department of Field Crops, Faculty of Agriculture, Van Yüzüncü Yıl University in 2019. The growth cabinet is approximately located at geographical coordinates of 8°33′47.4156″ N and 43°17′1.8672″ E.

The study was conducted in a completely randomized design (CRD) with 4 replications. The safflower seeds were sterilized with 5% sodium hypochlorite (NaClO) solution for 15 min and then washed with distilled water. Three seeds per pot were planted in plastic pots (2 L), filled with 1/3 sand, 1/3 perlite, and 1/3 soil mixtures. After germination, the best seedling was kept, and the other two were removed. The pots were placed in a plant growth room at 25 °C, 65% of humidity, and with a light/dark photoperiod of 16/8 h. The fertilization was homogeneously applied to all pots and contained 250 mg kg^−1^ N, 100 mg kg^−1^ P, and KH_2_PO_4_. Then cadmium was applied with a 1:5 Hoagland solution (pH 7) at five doses of 0 (control-Cd0), 25(Cd-25), 50 (Cd-50) and 75 (Cd-75) mg kg^−1^ in the form of (CdSO_4_)_3_.8H_2_O. The Cd applications were started 5 weeks after sowing (on day 35). The experiment was finished by harvesting on day 50 after germination (7th week).

### 2.3. Parameters Measurement

Growth traits, including plant height (cm), root length (cm), root and stem fresh weights (g), number of leaves, and leaf weight (g), were measured at crop harvest. Shoots and roots were separated from the harvested plants. The above-ground portion of each plant was split into two parts. One was used for mineral analysis. The other was kept in a deep freezer at −80 °C for the estimation of malondialdehyde (MDA) and ascorbate peroxidase (APX) activities. Lipid peroxidation in plants is expressed as MDA activity.

Mineral content such as potassium (K), calcium (Ca), magnesium (Mg), sodium (Na), chromium (Cr), copper (Cu), iron (Fe), manganese (Mn), nickel (Ni), lead (Pb), selenium (Se), zinc (Zn) and Cd was determined according to Lutts et al. [18]. The first three leaves were detached, placed in labeled glass jars, and stored in a deep freezer at −40 °C until analysis. For the analysis, 200 mg of each leaf sample was weighed and mixed with 10 mL of 0.1 N nitric acid (HNO_3_). The samples were kept in darkness at room temperature for one week in plastic boxes, then were shaken for 24 h. The K^+^ and Ca^+2^ ion concentrations were determined from the flame photometer’s prepared extract (Eppendorf, Germany) [19]. The analysis of Mg and Na was carried out by the wet burning method and determined with an atomic absorption spectrometer (AAS) [18].

The MDA activity was determined from leaves. The sample (0.5 g) was homogenized with 10 mL of 0.1% trichloroacetic acid (TCA). The mixture was centrifuged at 15,000× *g* for 5 min. Then, 1 mL of the centrifuged supernatant was mixed with 0.5% thiobarbituric acid (TBA) and dissolved in 4 mL of 20% TCA. The mixture was kept in a water bath at 95 °C for 30 min, then cooled rapidly in an ice bath and centrifuged again at 10,000× *g* for 10 min. Then, the absorbance of the supernatant was determined at 532 nm and 600 nm wavelengths. The malondialdehyde (MDA) activity was determined with the following formula [20]:

MDA (nmol ml^−1^) = [(A_532_ − A_600_)/155000] × 10^6^ (1)

The APX activity was estimated by grinding 0.5 g of the leaf sample in a porcelain mortar containing liquid nitrogen. The ground sample was homogenized with 10 mM phosphate buffer solution (pH 7.6) containing 50 mM of 0.1 mM Na-EDTA. The homogenized samples were centrifuged at 15,000 rpm for 15 min, and the fluids obtained were kept at 4 °C and used for enzyme measurement using the spectrophotometer. The APX activity was measured based on the cleavage rate of H_2_O_2_ at 290 nm (E = 2.8 mM cm^−1^) following the protocol of Sairam and Saxena [20].

### 2.4. Statistical Analysis

The analysis of variance (one-way ANOVA) was carried out on recorded data, and the averages were compared using Duncan’s multiple range test (DMRT) at *p* < 0.05. The graphs were made in Excel.

## 3. Results

### 3.1. Effect of Cd Doses on Morphological Attributes of Safflower

The Cd doses had a significant (*p* < 0.05 and *p* < 0.01) effect on the morphological attributes of safflower (Table 1). Plant height (PH) varied between 26.7 cm and 30.0 cm (with an average of 27.80 cm) after application of different Cd doses. The tallest plants were observed in the control treatment (Cd-0), which was statistically similar to Cd-25 and Cd-50 treatments. The highest Cd doses of 75 and 100 mg/L (*p* < 0.05) significantly decreased plant height by 3.49% and 6.64%, respectively.

The root length of safflower varied between 22.5 cm to 24.7 cm (an average variation of 23.7 cm) with Cd application (Table 1). The highest root length was noted at the control treatment (Cd-0), statistically on par with Cd-25 and Cd-50 treatments. The root length of safflower was significantly (*p* < 0.05) decreased (by 4.17% and 6.25%) with Cd doses of 75 and 100 mg/L, respectively).

Root fresh weight (RFW) varied in the range of 0.68 g to 1.32 g (an average of 0.98 g) with different Cd doses. The highest root fresh weight was observed with Cd-0, which was statistically similar to the Cd-25 treatments. The highest Cd dose (100 mg/L) significantly decreased fresh root weight by 48.48% compared to Cd-0 treatments.

Stem fresh weight varied between 0.99 g to 1.12 g (an average of 1.03 g) with Cd doses (Table 1). The highest stem fresh weight was found in the control treatment (Cd-0), which was statistically not different from the Cd-25 treatments. The lowest stem fresh weight of safflower was observed at 75 and 100 mg/L, which decreased the fresh stem weight of safflower by 6.60% and 14.15%.

Leaf fresh weight (LFW) and leaf numbers (LN) of safflower followed similar patterns, which were significantly influenced by Cd doses (Table 1). The LFW and LN varied in the range of 1.96 g–3.29 g and 17.7 g–19.5 g, respectively (an average variation of 2.60 g and 18.7). The highest values of LFW and LN were observed in control treatments (Cd-0), which were statistically similar to Cd-25 treatments. Application of Cd at a dose of 100 mg/L decreased LFW and LN indices by 40.42% and 9.23%, respectively, compared to the control treatment (Cd-0).

### 3.2. Effect of Cd Doses on Biochemical Attributes of Safflower

**Figure 1:**
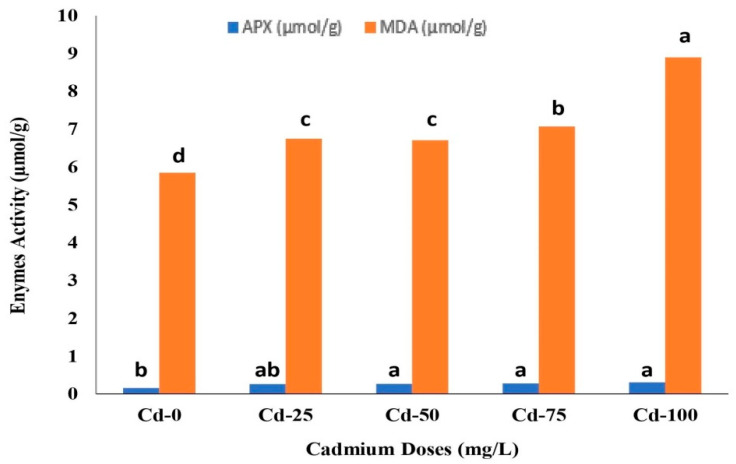
The effect of cadmium (Cd) doses on the biochemical parameters of safflower. The bars indicated with the same letters were not significantly different from each other on the basis of the Duncan multiple range test (DMRT) at *p* < 0.05. APX: Ascorbate peroxidases, MDA: Malondialdehyde.

Different doses of Cd had a significant (*p* < 0.01) influence on the biochemical attributes of safflower (Figure 1). The ascorbate peroxidase (APX) content ranged from 0.16 µmol/g to 0.31 µmol/g under the range of Cd doses. The highest APX activity was observed with the application of 100 mg/L Cd, which was statistically similar to the Cd doses of 50 and 75 mg/L. The APX activity increased by 93.75% under a Cd dose of 100 mg/L compared to control treatments (Cd-0).

The malondialdehyde (MDA) activity of safflower varied between 5.85 µmol/g and 8.90 µmol/g (Figure 1). The highest MDA activity was observed with 100 mg/L of Cd compared to control treatments (Cd-0). The treatments with Cd-100 increased MDA activity by 52.14% compared to Cd-0 (control) treatments. The lowest MDA activity was noted with control treatments.

### 3.3. Effect of Cd Doses on Macro and Micro Element Content of Safflower

The results showed that Cd doses and plant tissues significantly (*p* < 0.01) affected the mineral elements content of safflower. The average of mineral element content measured in different plant tissues (leaves, stems, and roots) showed a significant difference at *p* < 0.01 (Table 2).

The plant parts’ calcium (Ca) content varied in the range of 8.48–14.63 g/kg (Table 2). The highest average Ca content was noted in the stem tissues of safflower, whereas the lowest was observed in the roots, which was statistically similar to the leaf Ca content. Potassium (K) content in the safflower plant parts varied in the range of 4.83 g/kg–28.75 g/kg (Table 2). The average K content in the roots of safflower increased by 495% compared to the K content in leaves. Magnesium (Mg) content in the plant parts of safflower varied in the range of 3.23 g/kg–11.43 g/kg (Table 2). The concentrations of Na, Cd, Cr, Cu, Fe, Mn, Ni, Pb, Se, and Zn in the roots of safflower were increased by 101%, 333%, 838%, 1477%, 640%, 577%, 840%, 566%, 173% and 2452%, respectively, when compared with other plant parts.

**Figure 2:**
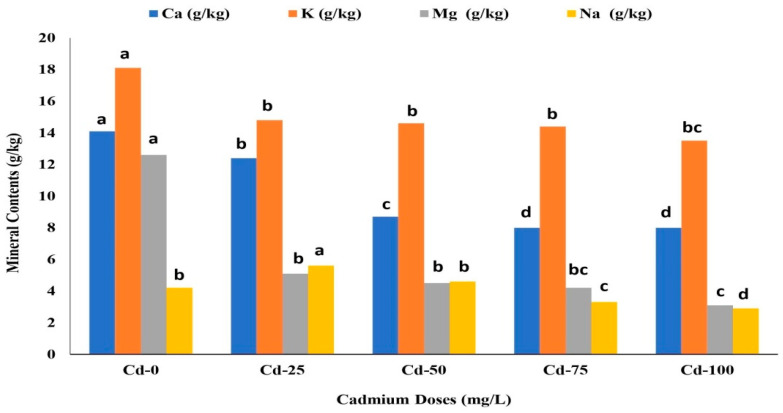
Effects of different cadmium concentrations on average calcium (Ca), potassium (K), magnesium (Mg) and sodium (Na) content of safflower. The similar letters above the bars do not show statistical difference at *p* < 0.05 based on the Duncan multiple range test (DMRT).

Different doses of Cd significantly influenced safflower’s macronutrient (Ca, K, Mg, and Na) content (Figure 2). The highest Ca, K, and Mg content in safflower plants was observed in control (Cd-0). The lowest Ca, K, and Mg content in safflower plants was recorded with 100 mg/L of Cd (Figure 2). Compared to the control, the Cd dose of 100 mg/L decreased the average Ca, K, and Mg content by 45.47%, 39.33%, and 79.28%, respectively. The highest Na average content was obtained with Cd-25. The lowest Na average content was observed in Cd-100 (Figure 2). Compared to Cd-25, applying 100 mg/L of Cd decreased the average Na content of safflower plants by 48.42%.

**Figure 3:**
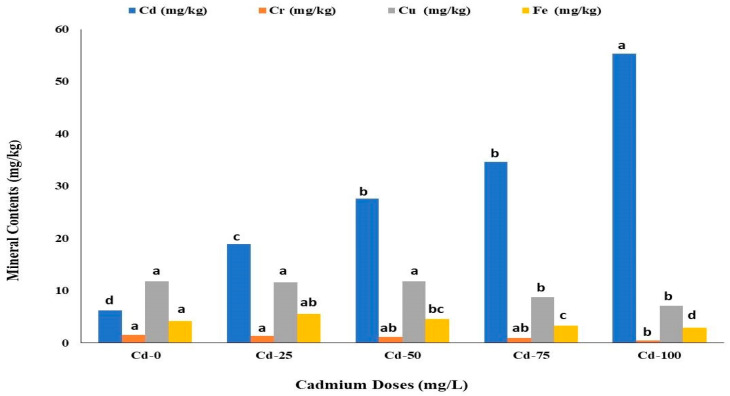
Effects of different cadmium doses on average cadmium (Cd), chromium (Cr), copper (Cu) and iron (Fe) content of safflower. The similar letters above the bars show no statistical difference at *p* < 0.05 on the basis of the Duncan multiple range test (DMRT).

Cadmium content in the safflower plant was significantly increased with Cd doses (Figure 3). The highest average Cd content was observed in Cd-100 treatments, whereas the lowest safflower Cd content was noted in Cd-0. From Cd-0 to Cd-100, an increasing trend of Cd content in the plant was observed. Compared to the control, applying 100 mg/L Cd increased the average Cd content of safflower plants by 89.42%.

The chromium content of safflower plants followed a decreasing trend with increasing Cd doses (Figure 3). The highest average Cr content was obtained in the control, which was statistically similar to the Cd-25. The lowest average Cr content was determined at a Cd dose of 100 mg/L, which decreased the average Cr content by 68.21% compared to the control.

The copper and iron content of safflower plants followed a decreasing trend with increasing Cd doses (Figure 3). The highest average Cu content was obtained in the Cd-0, which was statistically not different from the Cd-25 and Cd-50 treatments. The lowest average Cu content was also observed in the Cd-100, which was statistically similar to the Cd-75 (Figure 3). Compared to the Cd-0, applying 100 mg/L Cd decreased the average Cu content by 37.06%. The highest Fe average content was observed in the Cd-0, whereas the lowest Fe content was noted in the Cd-100. Compared to the control, the 100 mg/L of Cd application decreased the average Fe content by 66.67% (Figure 3).

**Figure 4:**
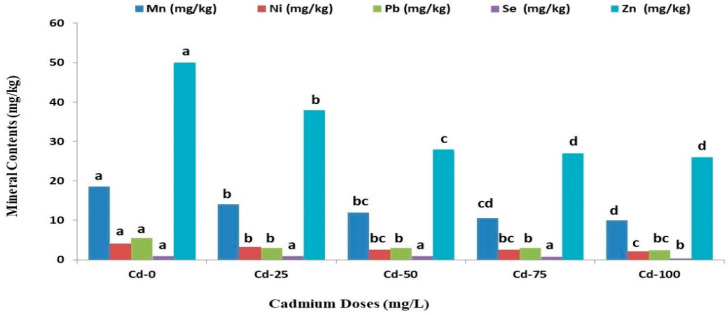
Effects of different cadmium concentrations on average manganese (Mn), nickel (Ni), lead (Pb), selenium (Se) and zinc (Zn) content of safflower. The similar letters above the bars show no statistical difference at *p* < 0.05 on the basis of the Duncan multiple range test (DMRT).

The content of Mn, Ni, Pb, Se, and Zn in safflower plants followed a similar trend with increasing Cd doses (Figure 4). The average content of these mineral elements decreased with increasing Cd doses. The highest content of Mn, Ni, Pb, Se, and Zn in safflower plants was found with the lowest Cd dose (Cd-0), while the lowest level of these minerals was observed with the Cd-100. Compared to the control, Cd application at a dose of 100 mg/L decreased the average Mn, Ni, Pb, Se, and Zn content by 45.62%, 50.38%, 54.37%, 33.33%, and 65.87%, respectively.

These results showed that the content of K, Na, Cd, Cr, Cu, Fe, Mn, Ni, Pb, Se, and Zn was higher in the roots than in other plant parts of safflower. In addition, the content of Ca and Mg was higher in stems than in other plant tissues (Table 2).

## 4. Discussion

Cadmium (Cd) is a toxic element to plants that alters nitrogen and carbohydrate metabolism [21], thus inhibiting enzyme activity and affecting stomatal conductance [22]. Plants exposed to soil Cd stress show leaf and root nitrate content [23], thus devastatingly affecting growth and physiological activities and polluting water and soil. Cadmium applications have increased in the last decades due to the high use of batteries, phosphorus fertilizers, and industrialization. Cadmium has been reported as a cause of soil and environmental pollution that adversely affects plant growth and development. In this context, the current study indicated that morphological growth traits of safflower were reduced with higher Cd doses compared to control treatments. This might be due to the negative effect of Cd on plant metabolism and oxidative damage which inhibits cell division and disrupts plant growth [24]. Several other studies also reported that higher Cd application decreased the growth traits of different plants [25,26]. The application of Cd to the plants’ roots disrupts the epidermal cells of the root surface. It causes the proliferation of plant stem cells that inhibit plant growth and development [27,28]. A cadmium stressed condition severely affects leaf weight, area, and the number of leaves [29], thus inhibiting the photosystem and decreasing plant morphological attributes.

Many studies have revealed that Cd has a toxic effect on plant functions, disrupting electron conductivity in photosynthesis, enzymatic activities, and carbohydrate metabolism in ways that affect host plants’ overall growth and development [30,31]. The present study showed that biochemical activity such as APX and MDA of safflower increased with increasing Cd doses (Figure 1). This increase might be due to the role of Cd stress in producing reactive oxygen species that leads to oxidative damage and disruption of cell structure. Several other studies on different crops reported that cadmium stress increases MDA and APX activities [32,33,34].

The content of mineral elements in safflower tissues (leaves, stem, and root) was affected by different Cd doses. Findings showed that all mineral element content of plants (i.e., Ca, K, Mg, and Na, Cr, Cu, Fe, Mn, Ni, Pb, Se, and Zn) decreased in response to Cd application. This decrease is possibly due to the inhibition of nutrient uptake and transportation under Cd stress, affecting photosynthesis and other nutrient mobility. Several other studies reported that increasing Cd stress decreased Ca and K uptake in different plants [32,35,36,37]. It has also been reported that Mg and Na content decreased with increasing Cd doses [38].

According to the results, the Cd content of the safflower increased with increasing Cd doses which is compatible with previous findings [37,39]. An overdose of Cd in the soil leads to hyper-accumulation of Cd in plant tissues, especially in roots, due to the compartmentation of Cd in the vacuoles [37,39].

There is a close relationship between the transport of Cu and nitrogen from old leaves that deteriorate plant color [40]. Thus, it has been proved in the current results that Cd stress decreased Cu content in safflower plants. In this study, the increasing of Cd doses decreased Fe/Zn content in safflower, which agrees with previous findings reporting competition between Cd and Fe/Zn over specific transporters in the root system, reducing the uptake of Fe/Zn through plant roots [41]. Regarding Mn content, this study showed that increasing Cd doses resulted in decreasing Mn content in plant tissues, and the lowest Mn content was determined in plant stems (Figure 4). The findings were similar to previous results [32,35,37,39]. This distinctive decrease in Mn content may be due to the competition of Mn with Cd for transport across the plasma lemma [42].

Regarding Ni, Pb, and Se content, this research showed decreases in these element in safflower tissues with increasing cadmium concentrations, which is different from previous results [32,43]. These differences may be due to the toxic effects of Cd on several plant functions at the physiological and biochemical levels from seed germination until maturation and final seed dispersal [44]. These adverse effects reduce crop nutrition values and inhibit storage protein catabolism and lateral root–formation. The reduction of transpiration, yield, uptake, and transportation of mineral elements has also been described [45].

## 5. Conclusions

Cadmium (Cd) is a relatively rare element and is not naturally found in a pure form. Considering the results of this study, Cd, even at low doses, has a toxic effect on safflower. Cadmium applications caused reductions in all studied morphological parameters. Especially compared to the control, the application of 100 mg/L of cadmium reduced plant height, root length, fresh root weight, stem fresh weight, fresh leaf weight, and leaf number by 11%, 8.91%, 48.48%, 18.75%, 40.43%, and 9.23%, respectively. The highest accumulation of Cd was detected in the roots of safflower. Increasing Cd doses affected growth and development negatively. The malondialdehyde and ascorbate peroxidase levels increased, indicating the presence of cell damage. The APX and MDA activities increased by 93.75% and 52.14% with increasing cadmium doses compared to control treatments. A gradual increase in Cd doses reduced mineral element content such as Ca, K, Mg, Na, Cr, Cu, Fe, Mn, Ni, Pb, Se, and Zn in leaves, stems, and roots of safflower as compared to control. Cadmium applications decreased the Ca, K, Mg, Cr, Cu, Fe, Mn, Ni, Pb, Se, and Zn content by 45.47%, 39.33%, 79.28%, 68.21%, 37.06%, 66.67%, 45.62%, 50.38%, 54.37%, 33.33%, and 65.87%, respectively, as compared to the control treatments. In terms of Na content, there was a 48.42% decrease as compared with using 25 mg/L of cadmium. As expected, there was an increase in the Cd content with increasing cadmium doses by 89.42% compared to the control treatment. Today, it is essential to investigate the damage levels and effects of heavy metal accumulation that threaten the environment and human health. Therefore, studies on achieving sustainable agriculture and production will help to take necessary precautions and measures against harmful substances. In addition, they will contribute to the literature in question by shedding light on valuable research in this field.

## Figures and Tables

**Table 1:** The effect of cadmium (Cd) doses on morphological parameters of safflower.

Cd Doses (mg/L)	Morphological Attributes					
	PH (cm)	RL (cm)	RFW (g)	SFW (g)	LFW (g)	LN
Cd-0	30.0 a	24.7 a	1.32 a	1.12 a	3.29 a	19.5 a
Cd-25	29.1 a	24.3 a	1.04 a	1.07 a	2.81 a	19.3 a
Cd-50	28.6 a	24.0 a	0.98 b	1.06 a	2.68 b	18.9 b
Cd-75	27.6 b	23.0 b	0.92 b	0.99 b	2.24 b	18.3 b
Cd-100	26.7 b	22.5 b	0.68 c	0.91 b	1.96 c	17.7 c
Average	27.8	23.7	0.98	1.03	2.60	18.7
Cd doses	*	*	**	*	**	**
CV (%)	4.35	14.6	18.7	17.3	18.9	10.3

* Significant at *p* < 0.05, ** Significant at *p* < 0.01. The data in columns with the same letters were not significantly different from each other, based on the Duncan multiple range test (DMRT). PH: Plant height, RL: Root length, RFW: Root fresh weight, SFW: Stem fresh weight, LFW: Leaf fresh weight, LN: Leaf numbers.

**Table 2:** The mineral elements average content in different plant tissues of safflower.

Plant Tissue	Mineral Elements												
	Ca	K	Mg	Na	Cd	Cr	Cu	Fe	Mn	Ni	Pb	Se	Zn
**Leaves**	8.59 b	4.83 c	3.23 c	3.02 b	13.59 b	0.29 b	1.66 b	181.94 b	4.24 c	1.11 b	2.01 b	0.54 b	3.21 b
**Stem**	14.63 a	8.62 b	11.43 a	2.79 b	14.27 b	0.56 b	2.77 b	52.08 c	6.38 b	0.68 b	1.05 c	0.45 b	3.90 b
**Root**	8.48 b	28.75 a	6.64 b	5.62 a	58.85 a	3.01 a	26.19 a	385.54 a	28.70 a	6.39 a	6.99 a	1.23 a	81.92 a
**Entire plant**	**	**	**	**	**	**	**	**	**	**	**	**	**
**Cd doses**	**	**	**	**	**	**	**	**	**	**	**	**	**

** Significant at *p* < 0.01. The data in columns with the same letters were statistically not different from each other on the basis of the Duncan multiple range test (DMRT). The Ca, K, Mg and Na values were measured as g/kg; The Cd, Cr, Cu, Fe, Mn, Ni. Se and Zn values were measured as mg/kg.

## Data Availability

Not applicable.
